# Population genetic data (COI, ddRAD) of *Sialislutaria* (Insecta, Megaloptera) from the Emscher catchment (Germany)

**DOI:** 10.3897/BDJ.13.e141997

**Published:** 2025-02-21

**Authors:** Martina Weiss, Florian Leese

**Affiliations:** 1 University of Duisburg-Essen, Essen, Germany University of Duisburg-Essen Essen Germany

**Keywords:** population genetic structure, gene flow, fragmentation, ddRAD, COI

## Abstract

**Background:**

In urban river systems, fragmentation of habitats and in-stream dispersal barriers play a major role in shaping the population genetic structure of freshwater macroinvertebrate species. In small, fragmented populations, effects of genetic drift and inbreeding are enhanced, which can lead to increased population differentiation and genetic diversity loss. One formerly strongly degraded and fragmented stream system in a highly urbanised area is the Emscher catchment in North Rhine-Westphalia, Germany. Major restoration efforts have led to an improvement of water and habitat quality over the past 20 years also in the formerly polluted tributaries, for example, the Boye catchment. However, the analysis of the population structure of two different amphipod and isopod species has revealed that some populations are still strongly isolated, indicating persisting gene flow barriers. In contrast, the effects are expected to be less pronounced in merolimnic species, which have an adult winged life stage, such as the alderfly *Sialislutaria* (Linnaeus, 1758) . However, this species was much less abundant in the Boye catchment and not found in adjacent catchments (only 9 of 41 sampling sites), reducing the power of possible analyses.

**New information:**

As no population genetic studies of *S.lutaria* have to our knowledge been published so far and genetic resources are generally scarce for this species, we generated and present here population genetic data for 70 *S.lutaria* specimens for the mitochondrial cytochrome c oxidase I (COI) gene and, more importantly, high resolution genomic single nucleotide polymorphism (SNP) data for 71 specimens, generated with double-digest restriction site-associated sequencing (ddRAD-seq). These data can be valuable for further studies, analysing the population genetic structure, dispersal pathways and potential gene flow barriers for *S.lutaria* on a larger geographic scale. Additional to presenting the data, we also give first insights in the population structure on a small geographic scale (area of approx. 15 km^2^). While the population differentiation was generally low, as expected on this small scale, we still found that gene flow was not equally strong between all populations, but that one population played a central role as a source and sink population, which cannot only be explained by the distance between populations.

## Introduction

The River Emscher catchment has been heavily impacted by urbanisation as it is located in one of the densest agglomerations in Europe, the “Ruhr Metropolitan Area” in the federal state of North Rhine-Westphalia in Germany (Gerner et al. 2018). The Emscher and part of its tributaries have been used as open sewer channels for almost a century until they were restored over the past 20 years (Gillmann et al. 2023). However, restoration is challenging, because of the strong pollution prior to restoration, the limited space for habitat development in the mostly heavily urbanised area, the few potential recolonisation source populations and the multiple persisting barriers for recolonisation (Gillmann et al. 2023, Winking et al. 2014). Despite restoration efforts, barriers between different streams or stream sections can still exist, leading to habitat fragmentation which is expected to increase genetic drift within the resulting small populations and reduce gene flow between populations (Miles et al. 2019). As a consequence, this can lead to increased genetic differentiation and reduced genetic diversity (Miles et al. 2019), reducing the adaptation capacity of populations and increasing the sensitivity to stress conditions which can, in the long run, affect the survival of keystone species (Bijlsma and Loeschcke 2012, Frankham 2005, Hughes et al. 2008, Švara et al. 2022). Potential dispersal and gene flow barriers can differ between freshwater macroinvertebrates depending for example on the species life history traits, i.e. if they are fully aquatic (hololimnic) or have a winged adult stage (merolimnic). While studies using population genetic data to estimate gene flow amongst populations have found that the population structure of hololimnic species is often high, with populations of different streams strongly differentiated already at low waterway distances, the opposite is often true for merolimnic species, where low differentiation has been found over large distances (e.g. Alp et al. (2012), Švara et al. (2022), Schröder et al. (2022), Weiss et al. (2022), Weigand et al. (2018)). However, especially in urban regions, also terrestrial migration barriers can exist for merolimnic species, which can lead to fragmentation.

In the overall project, we therefore aimed to analyse and compare the population genetic structure of different hololimnic species (i.e. *Asellusaquaticus*, *Proaselluscoxalis*, *Gammaruspulex* and *Gammarusfossarum*) which differ in their pollution tolerance and dispersal capability and the merolimnic alderfly *Sialislutaria* in different tributaries of the Emscher catchment, mainly Berne and Boye catchment. For all species, we sequenced the barcoding fragment of the cytochrome c oxidase I (COI) gene to check for the presence of potential cryptic species and obtain an overview over the population structure. Further, we generated high-resolution genome-wide SNP data using ddRAD-seq (double digest restriction site-associated DNA sequencing; Peterson et al. (2012)) to analyse contemporary gene flow patterns in more detail and identify persisting gene flow barriers and recolonisation pathways. While the amphipod and isopod species were found at many of the chosen 41 sampling sites and the data are presented in different publications (i.e. Weiss et al. accepted, Weiss et al. in preparation), *S.lutaria* was found in larger numbers only at five sampling sites (four additional sites with one or two specimens, each), which were all located in the Boye or its tributaries. To our knowledge, no population genetic study assessing gene flow and connectivity patterns of *S.lutaria* exists and, besides, even though the genome of this species has been published this year (Farr et al. 2024), only few genetic data are available for this species in general (e.g. Haring and Aspöck (2004)). Therefore, even though the dataset was too small to really address population genetic questions, we decided to still process the sampled specimens together with the amphipods and isopods to provide high quality genetic data, which can be useful, for example, for connectivity studies for this species on a larger scale, especially in combination with the now published genome.

## General description

### Purpose

This dataset provides first insights into the small-scale population genetic structure of *S.lutaria* in the heavily urbanised and recently restored Boye catchment, which is part of the Emscher catchment in the Ruhr Metropolitan Area in western Germany. The generated COI, but especially the provided high-resolution ddRAD data, hold particular value for analyses of the population structure of this genetically understudied species on a larger scale. They can be used to gain more knowledge on the realised gene flow of this species in urban and rural areas.

## Project description

### Study area description

The Emscher is a right tributary to the river Rhine and has a catchment area of 775 km^2^. It has several larger tributaries, for example, the Boye with a catchment area 75 km^2^, in which most of the sampling sites were located. While in the overall project, also sites in adjacent catchments were sampled (41 in total) which are described in detail in Weiss et al. (accepted), *S.lutaria* was only found in nine sites in the Boye catchment.

### Funding

The project was funded by a Forschungsgeist-Fellowship of the Stemmler-Stiftung within the Deutsches Stiftungszentrum (DSZ) (T0206/30095/2017) and supported by Collaborative Research Center (CRC) RESIST funded by the Deutsche Forschungsgemeinschaft (DFG, German Research Foundation) 1439/1—project number: 426547801.

## Sampling methods

### Sampling description

The sampling took place in 2019 and 2020 and in both years, larvae of *S.lutaria* were sampled using sieves and kick-nets. Specimens were then preserved in 96% denatured ethanol and stored at 4°C until further processing. *S.lutaria* was found at one near-natural and eight restored sampling sites (Table 1). At the near-natural site, BO23, located in the Schöttelbach, up to nine specimens were analysed in both years. Similarly abundant was the species at the restored Boye upstream site BO25 (restored 2009), where up to ten specimens were analysed. The other two sites, where *S.lutaria* was found in both years, were BO11 (Kirchschemmsbach, restored 2007, up to eight specimens) and BO13 (Haarbach, restored 2011, up to six specimens). At the two other sites located more upstream in both, the Kirchschemmsbach (BO12) and the Haarbach (BO14), two specimens were found only in 2019, in each. In 2020, nine specimens were found at BO15 (Vorthbach), but not in 2019. At two further restored sites in the Boye (BO20 and BO21) downstream of BO25, only one specimen was found and only in one sampling year, each.

### Step description

1. Prior to DNA extraction, *S.lutaria* larvae were morphologically identified and individually labelled leading to a total number of 78 specimens.
2. Depending on the sampling success, we extracted the DNA of 1 - 10 specimens per site. For DNA extraction, we used four to six legs per specimen and a small piece of muscle tissue from the body. While we used a salt precipitation protocol as described in Weiss and Leese (2016) in 2019, DNA extraction was conducted on a Biomek FX^P^ liquid handling workstation (Beckmann Coulter, Bread, CA, USA) in 2020 with a modified version of the bead-extraction protocol of the NucleoMag Tissue kit (Macherey-Nagel, Düren, Germany), which is described in detail in Buchner et al. (2021).
3. For all specimens, the barcoding fragment of the mitochondrial COI gene was amplified using the standard primers HCO2198 and LCO1490 (Folmer et al. 1994) as described in Weiss et al. (accepted) with small differences between the 2019 and the 2020 protocol. While species identification was possible with the obtained COI sequences for all specimens, the sequence quality was not sufficient for haplotype determination for eight specimens (Suppl. material 1).
4. For most of the specimens (except BO14, because DNA quality was too poor), we generated ddRAD-seq libraries according to the protocol described in Hupało et al. (2023). Details on sample preparation for each individual are given in Suppl. material 1. Depending on the initial DNA concentration, 28 to 500 ng DNA were used for the double digestion with the FastDigest restriction enzymes *Csp6*I and *Pst*I (Thermo Fisher Scientific). To choose the most suitable restriction enzymes and calculate adapter quantities for ligation (Peterson et al. 2012,) expected cut frequencies and number of fragments were estimated by *in silico* digestion using the script genomecut.pl (Rozenberg, https://github.com/evoeco/radtools/). As a reference, Illumina sequencing data (Accession number SRX8046471) were used, resulting in average cut frequencies of 297 bp for *Csp6*I and 8945 bp for *Pst*I. After measurement, samples were equimolarly pooled into one library, aiming at 40 ng DNA per specimen. For two specimens, the library preparation was not successful, resulting in 74 specimens which were paired-end sequenced on one HiSeq X Illumina lane (read length 2 x 150 bp, Macrogen, Korea) together with 19 *Gammarus* specimens.
5. For COI data analysis, sequences were assembled and edited in Geneious Prime 2022.0.2 (https://www.geneious.com). They were aligned with MAFFT 1.4.0 (Katoh and Standley 2013) as implemented in Geneious with default settings. To check species assignment, sequences were compared with the NCBI database (NCBI Resource Coordinators 2018). Sequences which were too short or had insufficient quality were excluded and the alignment cropped to the length of the shortest sequence per alignment. Then, COI haplotype distances and their frequencies were calculated and visualised in a minimum spanning network (Bandelt et al. 1999) in Popart v.1.7 (Leigh and Bryant 2015). Sequences of the resulting five haplotypes were uploaded to GenBank and are available under accession numbers PQ285806-PQ285810. Further, a map for visualisation of sampling sites and haplotype distribution was generated with a stream layer provided by the federal state authority LANUV (Gewässerstationierungskarte des Landes NRW © LANUV NRW (2013)) in QGIS v. 2.14.14 (http://qgis.org) and edited with Adobe illustrator 2024.
6. Pre-processing of ddRAD-seq data was similar to Weiss et al. (2018) including demultiplexing and removing of PCR duplicates. Raw data, as well as preprocessed data are available as an NCBI BioProject with the accession number PRJNA1163041 and as BioSamples with individual accession numbers given in Suppl. material 1. To identify loci and genotypes, the denovo_map.pl of Stacks v.1.34 (Catchen et al. 2013) was used with eight different parameter settings. The following analyses were conducted using the same Snakemake (Köster and Rahmann 2012) workflows as in Weiss et al. (2018) and Weiss et al. (accepted). To identify the most likely number of genetic clusters, individual ancestry coefficients were estimated, based on sparse non-negative matrix factorisation algorithms (sNMF; Frichot et al. (2014)) using the R-package LEA (Frichot and François 2015). Further, to analyse differentiation between populations, pairwise F_ST_ values (after Weir and Cockerham (1984)) between sites were calculated and significance was tested by bootstrapping over loci (1000 replicates, 0.025/0.975 confidence intervals) with the R-package hierfstat. To assess and visualise directional relative migration rates, we estimated G_ST_ (after Nei (1973)) for populations with n > 4 with the divMigrate function (Sundqvist et al. 2016) of the R-package diveRsity (Keenan et al. 2013).

## Geographic coverage

### Description

Boye catchment belonging to the Emscher catchment which is located in the Ruhr Metropolitan Area in western Germany.

### Coordinates

51.542215 and 51.579125 Latitude; 6.908860 and 6.960856 Longitude.

## Taxonomic coverage

### Description

This dataset contains genetic data (COI, SNP data) for *Sialislutaria*, Megaloptera.

### Taxa included

**Table:** 

Rank	Scientific Name	Common Name
species	*Sialislutaria*	alderfly

## Temporal coverage

**Data range:** 2019-3-29 – 2019-4-08; 2020-4-20 – 2020-4-22.

## Usage licence

### Usage licence

Creative Commons Public Domain Waiver (CC-Zero)

## Data resources

### Data package title

ddRAD raw data and demulitplexed and filtered data for each specimen of the whole project including *S.lutaria*, *G.fossaurm*, *G.pulex*, *A.aquaticus* and *P.coxalis*. For individual accession numbers for *S.lutaria*, see Suppl. material 1.

### Resource link

https://www.ncbi.nlm.nih.gov/sra/PRJNA1163041

## Additional information

### Insights into the population genetic structure of S.lutaria

**Figure 1.**
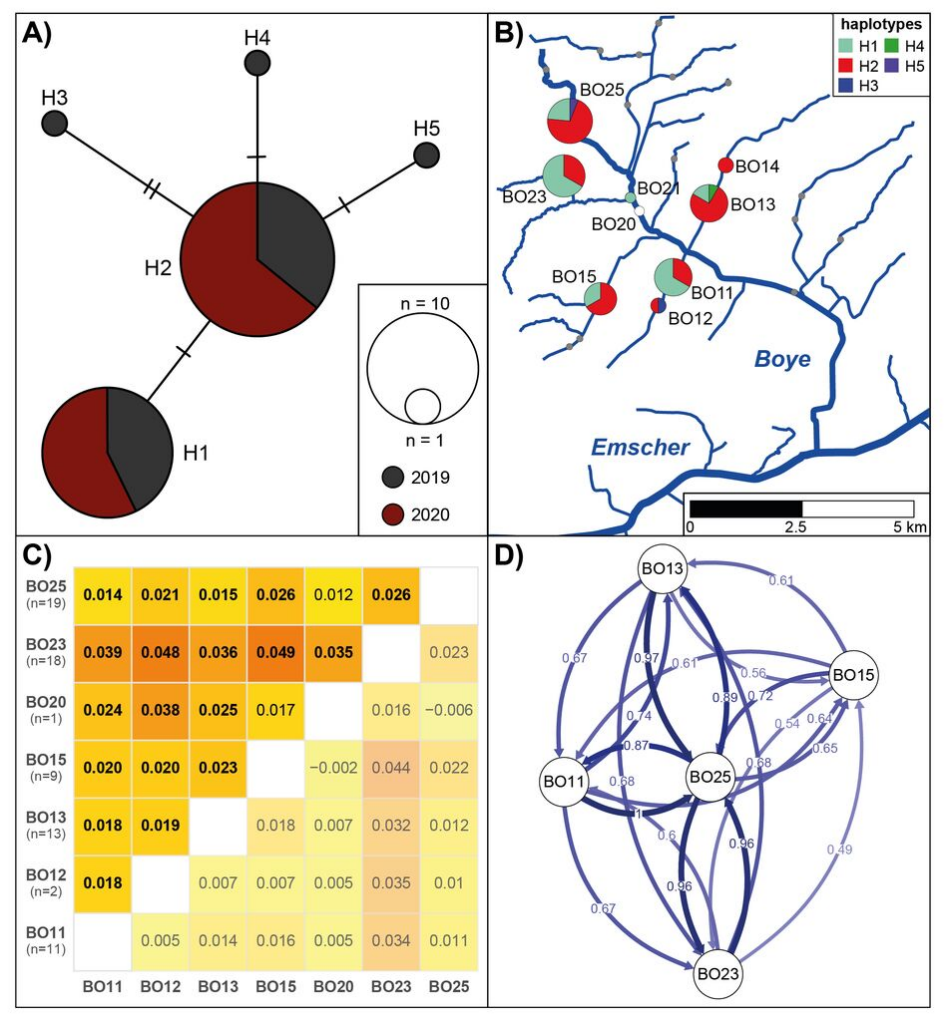
Population structure of *S.lutaria* in the Boye catchment. **A** COI minimum spanning network coloured according to sampling years. Vertical lines indicate mutations between haplotypes; **B** COI haplotype map showing the haplotype composition per site with sizes of pie charts scaled according to the numbers of sequences per site; **C** F_ST_ heat maps for the ddRAD data. Pairwise F_ST_ values are given above and the lower confidence intervals below the diagonal (values > 0 indicate significant differentiation, indicated in bold); **D** Relative migration network (G_ST_) for the ddRAD dataset.

The final COI alignment included 70 specimens and had a length of 560 bp, containing five variable sites, which were all synonymous substitutions. In total, five different haplotypes were detected, of which two (H1 and H2) were common at all larger sampling sites, while the other three were only found in one specimen each and only in 2019 (Fig. 1A and B).

The ddRAD sequencing was successful for 71 of the 76 specimens and resulted in 4686 loci shared by at least 90% of the specimens. While the sNMF analysis indicated the presence of only one cluster, all populations (except BO20, n = 1) were significantly differentiated from each other, even though differentiation was low (Fig. 1C). Values for migration rates (G_ST_, DivMigrate analysis) were generally high, but differed between sites (Fig. 1D). Highest values were detected between the upper Boye site BO25 and all other sites with lowest rates to BO15, even though the air distance was closer than to, for example, BO13, where migration rates were higher. Between all other sites, migration rates were lower.

Even though not many sites and specimens could be analysed for *S.lutaria* and the population structure was generally low as expected over these short geographic distances (0.3 to 4.6 km air distance, 0.4 to 6.6 km waterway distance) for species with adult winged live stages, high resolution genomic data revealed that gene flow is not equally strong between all sites and not directly correlated to the distance between sites. Interestingly, the most upstream Boye site, which was restored in 2009 (BO25) was detected as a central node from and to which most of the gene flow occurred (i.e. a source and sink population) and not BO23 which is the only site located in a near-natural stream section. To understand the drivers of gene flow patterns, more data would be needed. However, the data presented here show that it would be interesting to further study connectivity in this species on small and larger geographic scales and that a high resolution can be obtained by using methods such as ddRAD already on a small geographic scale despite a generally high connectivity.

## Supplementary Material

6F45692D-AA6C-5007-A640-E40A0291CA20 10.3897/BDJ.13.e141997.suppl1 Supplementary material 1 Information on ddRAD library preparation per sample, COI haplotype and NCBI accession numbers Data type Laboratory data Brief description Information on ddRAD library preparation per sample, i.e. used adapter combinations and PCR success. Further, COI haplotype and NCBI accession numbers for the haplotype and the ddRAD BioSample are given. For specimens, where no ddRAD library was generated, COI haplotype information is also given. File: oo_1151397.xlsx https://binary.pensoft.net/file/1151397 Martina Weiss, Florian Leese

## Figures and Tables

**Table 1.** Sampling sites with coordinates (WGS84), stream name (all sites Boye/Emscher catchment), ecological state and number of successfully analysed specimens per genetic marker.

**site**	**coordinates**		**stream name**	**ecological state**	**COI**		**ddRAD**	
	**latitude**	**longitude**			**2019**	**2020**	**2019**	**2020**
BO11	51.547875	6.943973	Kirch-schemmsbach	restored 2007	4	8	4	7
BO12	51.542215	6.939166	Kirch-schemmsbach	restored 2007	2	0	2	0
BO13	51.562628	6.955543	Haarbach	restored 2011	6	6	6	5
BO14	51.570292	6.960856	Haarbach	restored 2011	2	0	0	0
BO15	51.543524	6.920588	Vorthbach	restored 2011	0	9	0	9
BO20	51.561190	6.932998	Boye	restored 2002	0	0	1	0
BO21	51.563604	6.930191	Boye	restored 2002	0	1	0	0
BO23	51.568118	6.908860	Schöttelbach	near-natural	7	8	9	9
BO25	51.579125	6.910851	Boye	restored 2009	8	9	10	9
				**Sum**	**29**	**41**	**32**	**39**
